# Adropin ameliorates behavioral seizures and the relevant neuroinflammation, oxidative stress, and neural damage in a rat model of pentylenetetrazole-induced seizure potentially by reducing the activation of NF-κB/IkB-α signaling pathway

**DOI:** 10.1007/s11011-025-01654-2

**Published:** 2025-06-13

**Authors:** Shaafah Namulodi, Ibrahim Ethem Torun, Fahri Bayiroglu, Mehmet Salih Kaya, Erkan Kilinc

**Affiliations:** 1 Department of Physiology, Medical Faculty, Ankara Yıldırım Beyazıt University, Ankara, Türkiye; 2 Department of Physiology, Medical Faculty, Bolu Abant Izzet Baysal University, Bolu, Türkiye; 3 Department of Physiology, Cerrahpasa Faculty of Medicine, İstanbul University-Cerrahpaşa, İstanbul, 34098 Türkiye

**Keywords:** Adropin, Seizures, Neuroinflammation, Oxidative stress, Cognitive function

## Abstract

This study aimed to investigate the effects of adropin on seizure activity, neuroinflammation, oxidative stress, and cognitive function in a rat model of pentylenetetrazole (PTZ)-induced seizure. Male Wistar rats were randomly assigned to six groups (*n* = 7/each group), as follows: control, PTZ, adropin (2 µg/kg or 10 µg/kg) + PTZ, L-NAME + adropin + PTZ, and valproic acid + PTZ groups. Anticonvulsant medicine valproic acid was administered as positive control. Non-selective nitric oxide synthase inhibitor L-NAME was administered together with adropin to elucidate whether adropin exerts its possible effects through the nitric oxide pathway. Behavioral epileptic seizures, biochemical markers of neuroinflammation and relevant pathway, oxidative stress, cognitive function and neural survival/damage were assessed. Adropin (10 µg/kg) reduced PTZ-induced seizure severity and duration, and mitigated cortical and hippocampal pro-inflammatory (IL-1β, IL-6, TNF-α and related transcription factors pNF-κB-p65 and pIκBα), oxidant (MDA) and neural damage (GFAP) markers while elevating anti-inflammatory (IL-10), antioxidant (SOD) and neural survival (BDNF) markers. Combining adropin and L-NAME also exhibited similar effects to adropin alone. In other words, blocking systemic nitric oxide production did not alter the effects of adropin. However, adropin did not significantly improve cognitive performance in the passive avoidance test. Valproic acid, as a positive control, reversed the PTZ-induced effects. These findings suggest that adropin exhibits anticonvulsant, anti-inflammatory, antioxidant and neuroprotective properties in PTZ-induced seizure model potentially through modulation of NF-κB/IkB-α signalling. Therefore, adropin may be a multi-faceted and promising agent in the prevention and management of epileptic seizures in the future.

## Introduction

Epilepsy is a complex neurological disorder characterised by recurrent spontaneous seizures. It affects approximately 70 million people worldwide. The restrictive nature of the disease adversely affects patients’ functional capacity and reduces their quality of life. Additionally, nearly 50% of patients with epilepsy experience difficulties in memory and overall cognitive functions (Novak et al. 2022). It is widely accepted that epileptic seizures result from shifts in the balance between the excitatory and inhibitory neuronal circuits in the brain to the excitatory side. However, the pathophysiological events underlying this imbalance are poorly understood. Accumulating evidence suggests that neuronal oxidative stress, neuroinflammation and neuronal damage are implicated in seizure generation (Pearson-Smith and Patel 2017; Li et al. 2023; Sanz et al. 2024). Proinflammatory cytokines such as IL-1β, IL-6 and TNF-α secreted by glial cells and neurons have been reported to contribute to neuronal hyperexcitability (Li et al. 2023). Likewise, excessive production of reactive oxygen species induces neuronal excitability and ultimately seizure development by increasing glutamatergic transmission and decreasing GABAergic transmission (Borowicz-Reutt and Czuczwar 2020). There are a wide range of medications available to treat epilepsy, but they do not provide complete cure. They also do not provide neuroprotection and prevent epileptogenesis (Borowicz-Reutt and Czuczwar 2020). Furthermore, approximately one-third of patients with epilepsy are resistant to current medicines. Thus, new treatment approaches that exhibit anticonvulsant effects through modulation of neuroinflammation and oxidative stress may be attractive alternatives.

Adropin, an endogenous peptide encoded by the energy homeostasis-related gene, acts through various mechanisms such as neuroprotection, antioxidant effect, and preservation of blood–brain barrier integrity (Jasaszwili et al. 2020; Gunraj et al. 2023). Adropin exhibited anti-inflammatory properties by reducing the levels of pro-inflammatory cytokines such as TNF-α and IL-6 and promoting M2 phenotype in macrophages (Ali et al. 2022; Sato et al. 2018). Adropin also decreased oxidative stress and neutrophil infiltration and improved cognitive function in experimental ischemic stroke models (Yang et al. 2021, 2023). Up to now, only one study has investigated the effects of adropin on seizure activity and reported its effect in reducing epileptiform activity in a penicillin-induced seizure model (Dogru et al. 2023). However, its effects and mechanisms of action on epileptic seizures in different models have not yet been investigated. We therefore explored the effects and possible mechanisms of adropin on seizures, neuroinflammation, oxidative stress and cognitive status in a rat model of PTZ-induced seizure.

## Materials and method

### Animals

In this study, 42 male Wistar albino rats (3-month-old) weighing 250–360 g were obtained from Bolu Abant İzzet Baysal University Experimental Animals Application and Research Center. Animal care procedures, such as housing, welfare, and feeding, were performed at the same centre according to the National Institutes of Health guidelines for the care and use of laboratory animals. The rats were fed standard pellet feed and tap water ad libitum during the experimental period. The rats were housed in individual cages at 20–25 °C under a 12-h light/dark cycle. Ethical approval was obtained from the Bolu Abant İzzet Baysal University Animal Research Local Ethics Committee (Decision no. 2023/26).

### Drugs and reagents

Pentylenetetrazole and valproic acid (VPA) were purchased from Sigma-Aldrich (Schnelldorf, Germany). Adropin was purchased from MedChemExpress (New Jersey, USA). Nω-nitro-l-arginine methyl ester (l-NAME) was purchased from Cayman Chemicals (Michigan, USA). ELISA kits for rat interleukin (IL)−1β, TNF-α, brain-derived neurotrophic factor (BDNF), glial fibrillary acidic protein (GFAP), malondialdehyde (MDA), and superoxide dismutase-1 (SOD1) were purchased from Elabscience (Houston, Texas, USA). ELISA kits for rat IL-6, IL-10, phospho-inhibitory subunit of NF-κBα (pIκBα), and nuclear factor kappa B p65 (NF-κB-p65) were purchased from ELK Biotechnology (Denver, Colorado, USA). All drugs were dissolved in normal saline and administered intraperitoneal route in a volume of 0.25 ml. Therefore, an equal volume of normal saline was administered as vehicle to the control group.

### Experimental groups

The rats were randomly distributed into six groups (n = 7). Each group received two intraperitoneal injections 30 min apart. The control group (Sal + Sal) received 0.25 ml normal saline and then 0.25 ml normal saline. The model group (Sal + PTZ) received 0.25 ml normal saline and then 50 mg/kg PTZ. AD1 + PTZ group received 2 µg/kg adropin and then 50 mg/kg PTZ. AD2 + PTZ group received 10 µg/kg adropin and then 50 mg/kg PTZ. L-NAME + AD2 + PTZ group received 50 mg/kg L-NAME and 10 µg/kg adropin together and then 50 mg/kg PTZ. The positive control group (VPA + PTZ) received 300 mg/kg valproic acid and then 50 mg/kg PTZ. To determine whether the nitric oxide (NO) pathway is involved in the effect of adropin, it was also administered together with a non-selective NO synthase inhibitor L-NAME. In seizure behavior experiments, 10 µg/kg dose of adropin was chosen to administer together with L-NAME, since high dose of adropin rather than its low dose was effective in reducing seizure score.

### Establishment of PTZ-evoked seizure model and evaluation of seizure behaviors

Seizures were induced by administration of a single dose of PTZ at 50 mg/kg (Danis et al. 2023; Kilinc et al. 2022; Torun et al. 2022) 30 min after administration of normal saline or the other drugs in experimental groups except for control group.

**Fig. 1:**
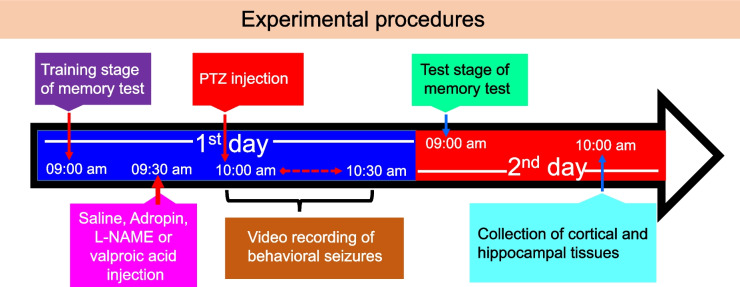
Schematic diagram of experimental procedures

Immediately after PTZ treatment, the rats were placed in plexiglass cages (40 cmx40cmx30cm) and seizure behaviours were observed and recorded for the first 30 min. The time taken to the first myoclonic jerk (FMJ), onset of the generalised tonic–clonic seizure (GTCS), and duration of the GTCS were recorded. The severity of the seizures was assessed using the modified Racine’s scale (Lüttjohann et al. 2009) as follows; stage 0, no response (no convulsions); stage 1, sudden cessation of behaviour/staring motionless; stage 2, facial twitching along the mouth area or eyes; stage 3, neck twitches (myoclonic twitches); stage 4, tonic–clonic seizure when the animal is in a sitting position; stage 5, tonic–clonic seizure with loss of righting reflex; stage 6, jumpy tonic–clonic seizure; stage 7, death. A schematic illustration of the time-course of experimental procedures is shown in Fig. 1.

### Recruitment of the passive avoidance test for assessment of cognitive behaviours

A step-through passive avoidance test, a behavioural learning and memory test (Eagle et al. 2016), was used to evaluate the cognitive status of the rats before and after treatment applications. The active and passive avoidance system device (MAY APAV-214 ELS, Commat Ltd., Ankara, Turkey) used had adjacent light and dark compartments with similar dimensions (26 × 26 × 30 cm) separated by a plate-like door that was automatically opened and closed using a software. The passive avoidance test had two phases, training and testing, and was conducted on two consecutive days. The training phase was performed on the first day of the experiments, 30 min before the first drug injections while the test phase was carried out after 24 h (Danis et al. 2023; Eagle et al. 2016). One hour after the retention test (24 h after injection of PTZ or its vehicle), cortical and hippocampal tissues were harvested.

### Training stage of cognitive behaviour experiments

The rat was placed in the light compartment and the door was opened five seconds later. The software automatically recorded the time taken to cross to the dark compartment immediately after the rat entered all four paws. Immediately after the rat entered the dark part, the door was closed, and a current shock of 1 mA was delivered to its feet for 5 s. After 30 s the rat was removed and returned to its cage. The training procedure was stopped if the rat did not enter the dark part for 120 s.

### Test stage of cognitive behaviour experiments

The test stage was performed 24 h after the training stage. A rat was placed in the light part of the device, and the door was opened 5 s later. The software automatically recorded the time taken to cross to the dark compartment for up to 300 s (step-through latency). No current shock was applied. If the rat did not enter the dark part within 300 s, the test was terminated, and the maximum score was noted as 300 s. Rats that did not exceed 300 s were considered as having learned. In this way, behaviours reflecting the memory status were scored.

### Time-course of experimental applications

The experimental application for all rats was performed at the same time points. On the first day of the experiment, at 09:00 am, the rats underwent the training stage of passive avoidance test. Treatment applications of adropin, L-NAME, or valproic acid were performed at 9:30 am. Seizures were induced by PTZ injection at 10:00 am. Immediately after PTZ injection, seizure behaviour was recorded for 30 min from 10:00 to 10:30 am. On the second day of the experiment, at 09:00 am, the rats underwent the retention test stage of the passive avoidance test. Following the test stage at 10:00 am, cortical and hippocampal tissues were harvested.

### Collecting the cortex and the hippocampus tissues from the brain

Immediately after the passive avoidance test stage, the rats were anaesthetised with ketamine (90 mg/kg, i.p.) and perfused intracardiac route with 150 mL PBS (pH 7.4). The cranium was then opened, the brain removed, and the cortex and hippocampal sections separated from the brain samples. Cortical and hippocampal tissues were weighed and homogenised using a tissue homogeniser (Light-duty Ultra-Turrax, ISOLAB, Wertheim, Germany) at 4 °C in a fixed volume of PBS (1 ml PBS:100 mg wet tissue ratio). The homogenates were centrifuged at at 10,000 g for 20 min at 4 °C. Supernatant samples were extracted and stored in eppendorf tubes at −20 °C until their IL-1β, IL-6, TNF-α, IL-10, pIκBα, NF-κB-p65, BDNF, GFAP, MDA and SOD1 contents were analysed. To avoid possible unfavourable effects of the waiting time period on ELISA measurements, the measurements were performed on all tissue homogenates on the 7th day following tissue homogenization.

### Biochemical analyses with ELISA

The concentrations of IL-1β, IL-6, TNF-α, IL-10, pIκBα, NF-κBp65, BDNF, GFAP, MDA and SOD1 were measured using enzyme-linked immunosorbent assay kits. The ELISA process was performed according to the manufacturer’s instructions for each kit. Following specific reagent applications and incubations, optical densities were quantified at 450 nm using an ELISA microplate reader (Epoch BioTek Instruments Inc., Winooski, VT, USA). The detection ranges were as follows: 31.25–2000 pg/ml for IL-1β, MDA, and BDNF, 7.82–500 pg/ml for IL-6 and IL-10, 15.63–1000 pg/ml for TNF-α, 0.16–10 ng/ml for pIκBα, NF-κB-p65, and SOD1, and 0.31–20 ng/ml for GFAP.

### Statistical analysis

SPSS for Windows (Ver. 22.0, Armonk, NY, US) was used for statistical data analysis. Data were exhibited as the mean ± standard deviation. The data were tested for normal distribution using the Shapiro–Wilk test. The seizure data did not exhibit normal distribution; therefore, the non-parametric Kruskal–Wallis test was used to compare the means of the groups then the difference between the two groups was determined by Mann–Whitney U test. Data from biochemical analysis were normally distributed; therefore, parametric one-way ANOVA test was used to compare the means of the groups followed by the post-hoc Tukey test. *P* value < 0.05 was considered significant.

## Results

### Adropin alleviated PTZ-induced behavioural seizures

No seizures were observed in the control group (Sal + Sal), therefore, only five groups were compared for the time to the first myoclonic jerk and seizure severity score. Moreover, no GTCS was observed in the VPA + PTZ group due to the efficacy of anti-epileptic medicine valproic acid; therefore, only four groups were compared for the onset and duration of GTCS. A significant difference was found among the groups for the latency to first myoclonic jerk (Chi square = 21.18, *p* < 0.001, df = 4), the latency to generalized tonic–clonic seizure (Chi square = 8.36, p = 0.039, df = 3), the duration of generalized tonic–clonic seizure (Chi square = 9.7, p = 0.021, df = 3) and the mean seizure score (Chi square = 22.2, *p* < 0.001, df = 4). Comparisons of all behavioral seizures parameters were performed with the Kruskal–Wallis H test.

**Fig. 2:**
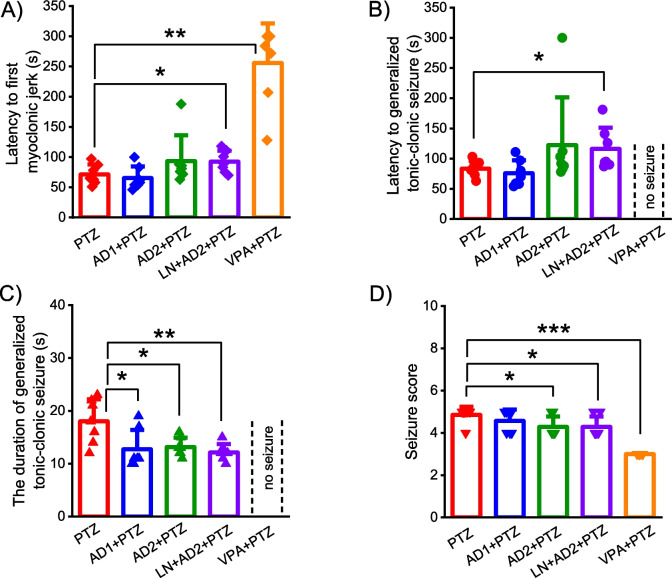
Anti-seizure effects of adropin in PTZ-induced epileptic conditions. Effects of adropin and other drug administrations on the latencies of first myoclonic jerk (**A**) and generalized tonic–clonic seizure (**B**), duration of generalized tonic–clonic seizure, (**C**) and seizure severity score (D). AD1, 2 µg/kg dose of adropin; AD2, 10 µg/kg dose of adropin; LN, L-NAME; PTZ, pentylenetetrazole; VPA, valproic acid. *p < 0.05, **p < 0.01 and ***p < 0.001

Adropin at a dose of 2 µg/kg significantly shortened the duration of GTCS compared to the model group (*p* = 0.025) but did not change the other seizure behaviours such as the time to the first myoclonic jerk and GTCS, and seizure severity score (*p* > 0.05, Fig. 2A-D). On the one hand, adropin at a dose of 10 µg/kg significantly shortened the duration of GTCS (*p* = 0.029) and lowered seizure severity score (*p* = 0.037) compared to the model group (Fig. 2C-D). Furthermore, adropin in combination with L-NAME significantly extended the onset of the first myoclonic jerk (*p* = 0,048) and GTCS (*p* = 0.025), and reduced the GTCS duration (*p* = 0.01) and seizure severity score (*p* = 0.037) compared to the model group (Fig. 2A-D). Valproic acid administered as a positive control fully suppressed GTCS (no seizure occurred) and significantly prolonged the onset of the first myoclonic jerk (*P* = 0.002), and reduced the seizure severity score (*P* = 0.0001) compared to the model group (Fig. 2A-D).

### Adropin inhibited the expression of NF-κB/IκBα and related pro-inflammatory cytokines and increased the expression of anti-inflammatory cytokine in the cortex and hippocampus

One-way ANOVA test revealed a significant difference between the groups in terms of the cortical and hippocampal concentrations of phospho-NF-κB p65 (cortex: F(5, 36) = 22.08, *p* < 0.001; hippocampus: F(5, 36) = 12.25, *p* < 0.001), pIκBα (cortex: F(5, 36) = 8.36, *p* < 0.001; hippocampus: F(5, 36) = 8.14, *p* < 0.001), IL-1β (cortex: F(5, 36) = 5.61, *p* = 0.001; hippocampus: F(5, 36) = 7.11, *p* < 0.001), IL-6 (cortex: F(5, 36) = 9.29, *p* < 0.001; hippocampus: F(5, 36) = 9.3, *p* < 0.001), TNF-α (cortex: F(5, 36) = 6.42, *p* < 0.001; hippocampus: F(5, 36) = 5.27, *p* = 0.001), and IL-10 (cortex: F(5, 36) = 10.39, *p* < 0.001; hippocampus: F(5, 36) = 15.42, *p* < 0.001).

**Fig. 3:**
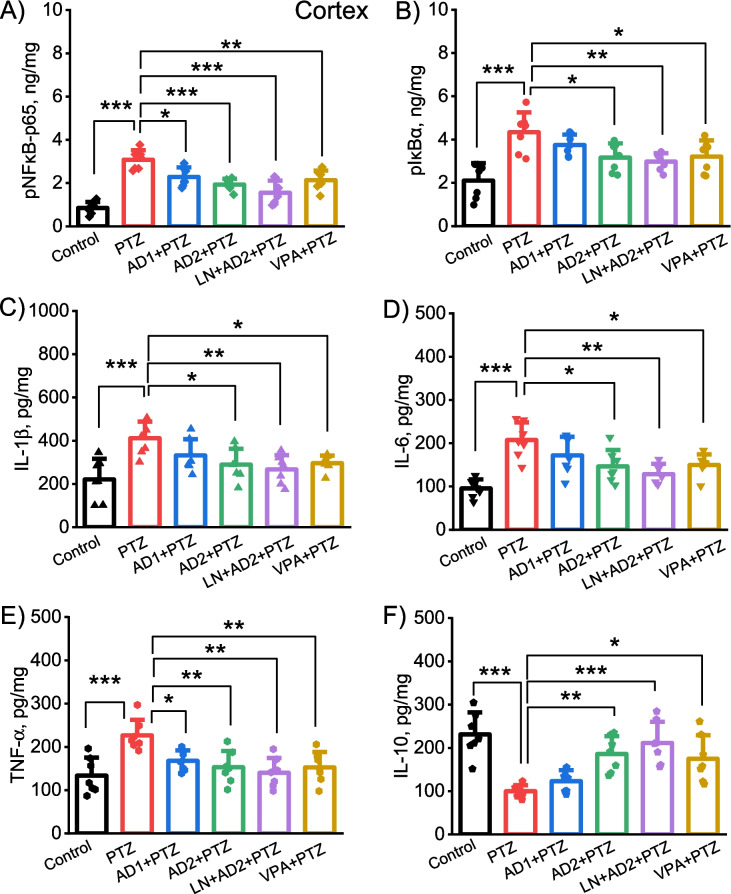
Anti-neuroinflammatory effects of adropin in the cortex in PTZ-induced epileptic conditions. Effects of adropin and other drug administrations on the levels of pNF-κB-p65 (**A**), pIκBα (**B**), IL-1β (**C**), IL-6 (**D**), TNF-α (**E**) and IL-10 (**F**) in the cortex. AD1, 2 µg/kg dose of adropin; AD2, 10 µg/kg dose of adropin; LN, L-NAME; PTZ, pentylenetetrazole; VPA, valproic acid. *p < 0.05, **p < 0.01 and ***p < 0.001

**Fig. 4:**
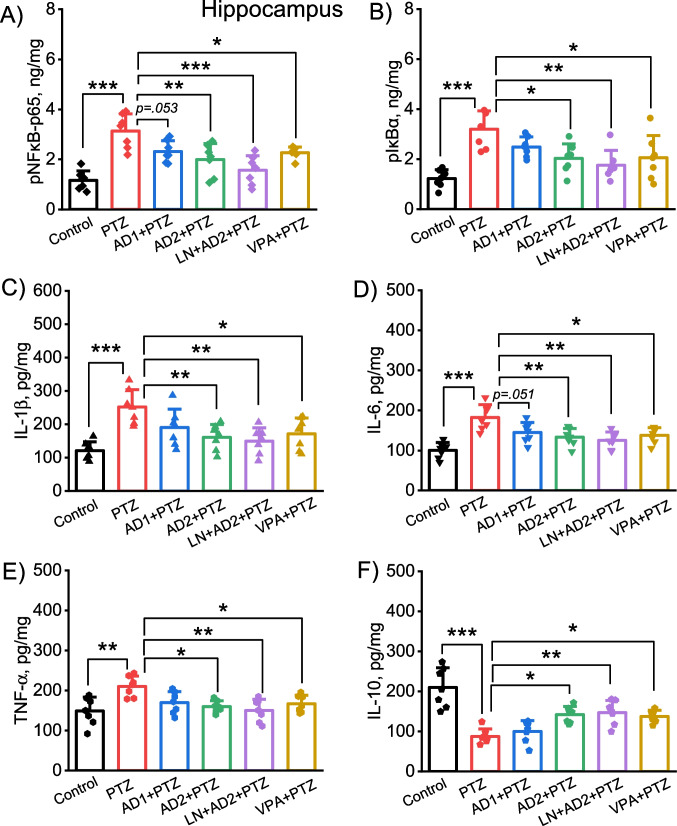
Anti-neuroinflammatory effects of adropin in the hippocampus in PTZ-induced epileptic conditions. Effects of adropin and other drug administrations on the levels of pNF-κB-p65 (**A**), pIκBα (**B**), IL-1β (**C**), IL-6 (**D**), TNF-α (**E**) and IL-10 (**F**) in the hippocampus. AD1, 2 µg/kg dose of adropin; AD2, 10 µg/kg dose of adropin; LN, L-NAME; PTZ, pentylenetetrazole; VPA, valproic acid. *p < 0.05, **p < 0.01 and ***p < 0.001

PTZ administration significantly increased both cortical and hippocampal concentrations of key molecules in the proinflammatory signaling cascade including the phospho-NF-κB p65, pIκBα, TNF-α, IL-1β, and IL-6 and significantly reduced cortical levels of anti-inflammatory cytokine IL-10 (*p* = 0,001 for hippocampal TNF-α, *p* = 0.0001 for the other biomarkers) compared to control group (Figs. 3A-F and 4A-F). Adropin at a dose of 2 µg/kg significantly reduced PTZ-induced elevation of cortical pNF-κB p65 and TNF-α, (*p* = 0.013, *p* = 0.037, Fig. 3A and E) and also alleviated PTZ-induced elevation of hippocampal pNF-κB p65 and IL-6 (*p* = 0.053, *p* = 0.051, Fig. 4A and D). However, the high dose of adropin (10 µg/kg) significantly mitigated PTZ-induced increases in both cortical and hippocampal pNF-κB p65, pIκBα, IL-1β, IL-6, TNF-α, and also significantly increased the decreased cortical and hippocampal levels of IL-10 (*p* = 0.0001, *p* = 0.032, *p* = 0.034, *p* = 0.017, *p* = 0.005 and *p* = 0.005 for cortical levels of biomarkers, respectively, *p* = 0.002, *p* = 0.014, *p* = 0.005, *p* = 0.004, *p* = 0.01and *p* = 0.014 for hippocampal levels of biomarkers, respectively, Figs. 3A-F and 4A-F).

Likewise, the combination of adropin with L-NAME attenuated PTZ-induced rises in both cortical and hippocampal pNF-κB p65, pIκBα, IL-1β, IL-6, TNF-α, and also significantly enhanced the reduced cortical and hippocampal levels of IL-10 (*p* = 0.0001, *p* = 0.0001, *p* = 0.007, *p* = 0.0001, *p* = 0.0001 and *p* = 0.0001 for cortical levels of biomarkers, respectively, Fig. 3A-F *p*= 0.0001, *p* = 0.001, *p* = 0.001, *p* = 0.001, *p* = 0.002 and *p* = 0.006 for hippocampal levels of biomarkers, respectively, Fig. 4A-F). Positive control valproic acid restored PTZ-induced changes in the inflammation-related biomarkers in both cortex and hippocampus (*p* < 0.05, Figs. 3A-F and 4A-F).

### Adropin restored the PTZ-induced alterations in the levels of oxidant/antioxidant biomarkers in both the cortex and hippocampus

According to one-way ANOVA test, there was a significant difference between the groups in terms of the concentrations of MDA (cortex: F(5, 36) = 8.18, *p* < 0.001; hippocampus: F(5, 36) = 6.48, *p* < 0.001) and SOD (cortex: F(5, 36) = 4.67, *p* = 0.002; hippocampus: F(5, 36) = 5.12, *p* = 0.001).

**Fig. 5:**
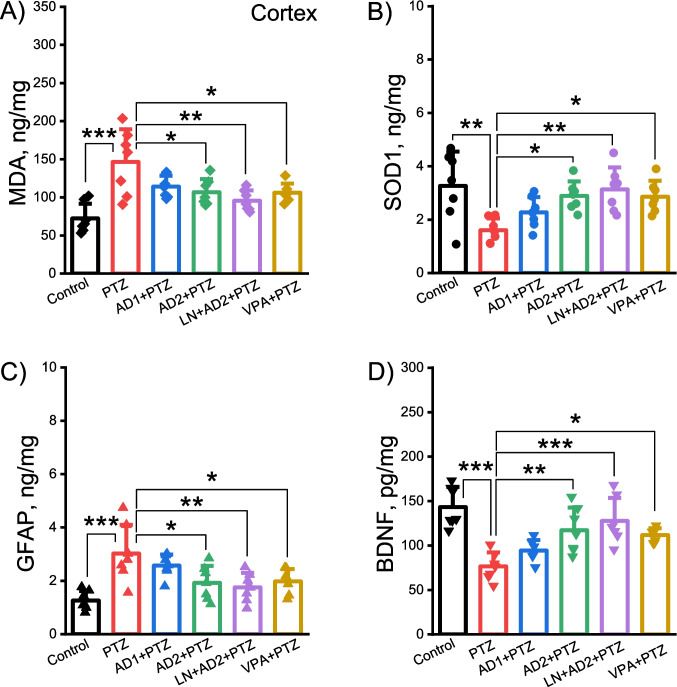
Antioxidant and neuroprotective effects of adropin in the cortex in PTZ-induced epileptic conditions. Effects of adropin and other drug administrations on the levels of MDA (**A**), SOD1 (**B**), GFAP (**C**) and BDNF (**D**) in the cortex. AD1, 2 µg/kg dose of adropin; AD2, 10 µg/kg dose of adropin; LN, L-NAME; PTZ, pentylenetetrazole; VPA, valproic acid. *p < 0.05, **p < 0.01 and ***p < 0.001

**Fig. 6:**
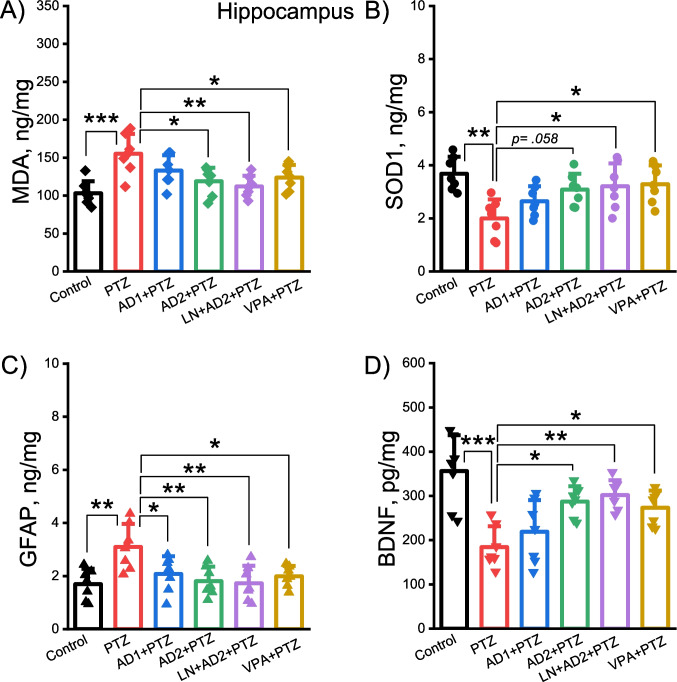
Antioxidant and neuroprotective effects of adropin in the hippocampus in PTZ-induced epileptic conditions. Effects of adropin and other drug administrations on the levels of MDA (**A**), SOD1 (**B**), GFAP (**C**) and BDNF (**D**) in the hippocampus. AD1, 2 µg/kg dose of adropin; AD2, 10 µg/kg dose of adropin; LN, L-NAME; PTZ, pentylenetetrazole; VPA, valproic acid. *p < 0.05, **p < 0.01 and ***p < 0.001

PTZ administration significantly elevated cortical and hippocampal levels of oxidative stress biomarker malondialdehyde (MDA) (*p* = 0.0001, Figs. 5A and 6A) and decreased cortical and hippocampal levels of antioxidant enzyme superoxide dismutase (SOD1) compared to control group (*p* = 0.003, *p* = 0.001, Figs. 5B and 6B). Adropin at a dose of 10 µg/kg significantly diminished PTZ-induced increases in levels of MDA (*p* = 0,023 for cortex, Fig. 5A, *p* = 0,012 for hippocampus, Fig. 6A), and enhanced the reduced levels of SOD1 in both cortex (*p* = 0,035, Fig. 4B) and hippocampus (*p* = 0,058, Fig. 6B) while adropin at a dose of 2 µg/kg was not effective (*p* > 0.05, Figs. 5A-B and 6A-B). Likewise, combining adropin and L-NAME restored the PTZ-induced alterations in the levels of MDA and SOD1 in both the cortex and hippocampus (*p* = 0.002 for cortical and hippocampal MDA, *p* = 0.008 for cortical SOD1, *p* = 0.025 for hippocampal SOD1, Figs. 5A-B and 6A-B). Valproic acid as positive control significantly ameliorated PTZ-induced changes in MDA and SOD1 levels in both the cortex and hippocampus (*p* = 0.021 and *p* = 0.038 for cortical and hippocampal MDA, *p* = 0.043 and *p* = 0.014 for cortical and hippocampal SOD1, Figs. 5A-B and 6A-B).

### Adropin ameliorated the PTZ-induced changes in the levels of memory- and damage-related biomolecules in both the cortex and hippocampus

Brain-derived neurotrophic factor (BDNF) is involved in the formation and consolidation of long-term memory. Glial fibrillary acidic protein (GFAP) serves as a biomarker of central nervous system damage. One-way ANOVA test showed that there was a significant difference between the groups in terms of the concentrations of BDNF (cortex: F(5, 36) = 10.38, *p* < 0.001; hippocampus: F(5, 36) = 8.91, *p* < 0.001) and GFAP (cortex: F(5, 36) = 6.86, *p* < 0.001; hippocampus: F(5, 36) = 4.84, p = 0.002). PTZ treatment significantly lowered BDNF levels and significantly elevated GFAP levels in both the cortex and hippocampus compared to control group (*p* = 0.0001 and *p* = 0.003 for cortical and hippocampal GFAP, Figs. 5C and 6C, Fig, *p* = 0.0001 for cortical and hippocampal BDNF, Figs. 5D and 6D). The 10 µg/kg dose of adropin (but not 2 µg/kg) significantly ameliorated cortical and hippocampal levels of GFAP and BDNF induced by PTZ (*p* = 0.026 and *p* = 0.05 for cortical and hippocampal GFAP, Fig. 5C and 6C p = 0.005 and *p* = 0.013 for cortical and hippocampal BDNF, Figs. 5D and 6D). Similarly, the combination of adropin with L-NAME significantly improved cortical and hippocampal levels of GFAP and BDNF induced by PTZ (*p* = 0.007 and *p* = 0.003 for cortical and hippocampal GFAP, *p* = 0.0001 and *p* = 0.003 for cortical and hippocampal BDNF, Figs. 5C-D and 6C-D). Positive control valproic acid significantly improved PTZ-evoked alterations in cortical and hippocampal concentrations of GFAP and BDNF (*p* = 0.039 and *p* = 0.027 for cortical and hippocampal GFAP, *p* = 0.021 and *p* = 0.043 for cortical and hippocampal BDNF, Figs. 5C-D and 6C-D).

### The effects of drug treatments on cognitive status

Analysis of the training phase of the passive avoidance test showed no statistically significant differences between the groups (Chi square = 2.43, *p* = 0.787, df = 5, Kruskal–Wallis H test). This suggests that the initial behaviour was consistent across all groups. The similarity in the time spent in the light compartment suggests that the baseline behaviour was comparable. In the test phase, although there were variations in the mean times across the groups, these differences were not statistically significant (Chi square = 6.33, *p* = 0.275, df = 5, Kruskal–Wallis H test). Notably, the PTZ group displayed a lower mean step-through latency (10.9 ± 2.9 s) compared to the control (66.8 ± 39.5 s), whereas the combination of adropin and L-NAME exhibited a higher step-through latency compared to the PTZ group (85.5 ± 44.4 s, Table 1). However, the lack of statistical significance does not allow them to be accepted as significant.

**Table 1:** Effects of adropin and other drug administrations on latencies to move to the dark compartment in the passive avoidance test

	The stage of passive avoidance test	Groups						*P* value
		Control	PTZ	AD1 + PTZ	AD2 + PTZ	LN + AD2 + PTZ	VPA + PTZ	
Latency to dark compartment	Training trial (first day of the experiment)	12.7 ± 4.2	12.9 ± 6.5	30.4 ± 14.7	5.4 ± 1.6	12.6 ± 5.3	8.7 ± 3.1	0.787^a^
	Test trial (second day of the experiment)	66.8 ± 39.5	10.9 ± 2.9	75.8 ± 38.6	58.9 ± 40.5	85.5 ± 44.4	59.4 ± 30.1	0.275^a^

^a^The means of the groups were compared using the Kruskal–Wallis H test, and no significant difference was found between the groups

*AD1* 2 µg/kg dose of adropin; *AD2* 10 µg/kg dose of adropin; *LN* L-NAME; *PTZ* pentylenetetrazole; *VPA* valproic acid

## Discussion

This study investigated the anti-seizure, anti-neuroinflammatory, antioxidant and neuroprotective effects of adropin in an acute model of PTZ-induced seizure. The major findings of the study were that adropin reduced the severity of PTZ-induced seizures and suppressed neuroinflammation by reducing activation of NF-κB/IkB-α signaling pathway, and relieving oxidative stress, and mitigating neuronal injury-related mediators.

Modulators of excitatory and inhibitory ion channels have long been used effectively to control epileptic seizures. However, the recurrence of epileptic seizures despite these drugs and the fact that the some patients are still resistant to treatment indicate that there are other pathological processes underlying the neuronal excitatory/inhibitory imbalance. Recent studies recognized that neuroinflammation and oxidative stress are two key contributors of the epileptogenesis (Borowicz-Reutt and Czuczwar 2020; Villasana-Salazar and Vezzani 2023). Persistent proinflammatory cytokines and reactive oxygen species encourage epileptogenesis by lowering seizure thresholds (Borowicz-Reutt and Czuczwar 2020; Villasana-Salazar and Vezzani 2023). Modulating the inflammatory and oxidative status in the brain and thus maintaining the excitatory/inhibitory balance may represent a new pathophysiology-focused treatment approach to control epileptic seizures and may also be promising for resistant patients. Based on this notion, we investigated the impacts of the peptide adropin, which stands out with its anti-inflammatory, antioxidant and neuroprotective properties, in the PTZ-induced seizure model.

In the current research, adropin shortened the duration of GTCS and reduced the seizure severity score. These findings clearly indicate that adropin acts an anticonvulsant. Recently a study has showed that adropin diminished the penicillin-evoked epileptiform activity in rats (Dogru et al. 2023). Our findings are in line with that study and provide further experimental evidence for the anti-epileptic potential of adropin. Moreover, our study demonstrates for the first time the suppressive effects of adropine on seizure behaviors.

The current study found that PTZ led to the elevated concentrations of inflammatory and oxidant biomarkers such as pNF-κB, pIkB-α, IL-1β, IL-6, TNF-α and MDA and the reduced levels of anti-inflammatory and antioxidant mediators such as IL-10 and SOD in both cortex and hippocampus. Our findings are consistent with previous studies reporting similar results in the PTZ-induced model (Danis et al. 2023; Torun et al. 2022; Kilinc et al. 2021; Cheng et al. 2024; Yu et al. 2021; Chen et al. 2017; Nkwingwa et al. 2023). Additionally, we chose the cortex and hippocampus for biomarker measurements since they are strategic brain structures related to the epileptogenesis (Skopin et al. 2020). On the other hand, we found that adropin restored the inflammatory and oxidative status impaired by PTZ through mitigating levels of pro-inflammatory cytokines IL-1β, IL-6 and TNF-α and oxidant biomarker MDA, and by enhancing anti-inflammatory cytokine IL-10 and antioxidant enzyme SOD in both cortex and hippocampus. Previous studies showed that adropin exhibited anti-inflammatory effects by reducing pro-inflammatory cytokines such as IL-1β, IL-6, TNF-α and inducible NOS in various inflammation-associated diseases/conditions (Ali et al. 2022; Wang et al. 2025). Likewise, adropin displayed antioxidant properties by mitigating oxidative stress products and by enhancing antioxidant capacity in different disorder conditions (Maurya et al. 2024; Sümer Coşkun et al. 2024; Chen et al. 2019). Additionally, adropin deficiency has been found to be associated with elevated oxidant status and proinflammatory cytokine levels in humans and rodents (Simac et al. 2022; Zorlu et al. 2022; Yang et al. 2018). Our findings are consistent with previous studies regarding the anti-inflammatory and antioxidant properties of adropin. However, our study demonstrates for the first time the anti-neuroinflammatory and antioxidant effects of adropine in a rodent model of seizure.

Additionally, we also measured the levels of pNF-κB and pIkB-α to reveal the possible signaling pathway mediating these effects of adropin. Our results showed that adropin reduced PTZ-induced the levels of pNF-κB and pIkB-α in both cortex and hippocampus. These findings suggest that adropin exerts its anti-neuroinflammatory effects by inhibiting the activation of the NF-κB/IkB-α signaling pathway in the PTZ-induced seizure model. Considering the role of neuroinflammation in the generation of epileptic seizures, it may be speculated that the suppression of the inflammatory NF-κB/IkB-α signaling pathway plays an important role in the anticonvulsant effects of adropin. On the one hand, the brain has a high fat content and its high energy demand necessitates more oxygen use than other tissues. This situation makes the brain more susceptible to oxidative stress and subsequent damage. Consistent with this, preclinical and clinical studies have reported increased oxidative stress markers in epilepsy conditions (Borowicz-Reutt and Czuczwar 2020). Thus, suppression of oxidative stress is important to prevent the occurrence of epileptic seizures. In the present study, adropin demonstrated antioxidant properties in epileptic conditions, further strengthening its anti-epileptic potential.

On the other hand, it is well established that memory is impaired in epilepsy patients (Novak et al. 2022) and in the PTZ-induced rodent model of seizure (Danis et al. 2023). Therefore, we employed the passive avoidance test and measured memory/memory impairment-related molecules to reveal the effect of adropin on PTZ-induced memory impairment. GFAP is key biomarker of neural damage and cognitive impairment (Bettcher et al. 2021; Leipp et al. 2024). BDNF is an important mediator associated with learning and memory (Miranda et al. 2019). PTZ administration elevated GFAP levels while reducing BDNF levels in the cortex and hippocampus. In addition to being a direct marker of astrocyte activation, GFAP is also widely evaluated an indirect marker of neuronal degeneration in specific brain regions such as the hippocampus in PTZ-induced seizure models (Tambe et al. 2016; Younis et al. 2024; Ahmed et al. 2005; El-Hefnawy et al. 2022). In the current study, it would be more useful if more precise methods such as immunohistochemical evaluation of GFAP and Nissl staining were used in addition to measuring GFAP by ELISA to evaluate neuronal damage. However, the increased GFAP concentrations in our study indicate, at least indirectly, that PTZ caused cortical and hippocampal neurodegeneration, and our findings are also consistent with previous studies (Tambe et al. 2016; Younis et al. 2024; Ahmed et al. 2005; El-Hefnawy et al. 2022). Reduced BDNF levels indicate impaired neuroprotection and potential cognitive deficits, as BDNF is crucial for neuronal survival, synaptic plasticity, and cognitive function (Khatoon et al. 2023; Sharma et al. 2020). This imbalance may contribute to seizure progression and cognitive impairments, highlighting the interplay between inflammatory and neuroprotective mechanisms in development of seizures. Adropin reduced the increased GFAP level and enhanced the decreased BDNF level due to PTZ effect. These findings imply that adropin may have neuroprotective and memory-improving effects. Our findings are consistent with the memory-enhancing effect of adropin by increasing BDNF levels in rat hippocampus (Ozkan et al. 2022). However, although adropin prolonged the step-through latency, this was not found to be statistically significant due to the high standard deviations of the values. This situation can be overcome with studies conducted with higher sample sizes, provided that they remain within ethical framework.

NO plays an important role in the modulation of seizure susceptibility. It is reported that NO may have pro- or anti-convulsant action, depending on the seizure model, the type of seizure-inducing pharmacological drug, its dose and the route of administration (Banach et al. 2011). Adropin activates the PI3K/Akt signaling pathway and Akt plays a key role in NO production (Rooban et al. 2024). Adropin-induced vasodilation was largely reversed by NO synthase inhibitor L-NAME, indicating the critical role of NO in mediating the effects of adropin (Fujie et al. 2021). Considering together the relevance of adropin in NO generation and NO in epileptic seizures, to elucidate whether adropin exerts its effects via NO, we also administered it together with non-selective NO synthase inhibitor L-NAME. In the current study, combining adropin and L-NAME did not reverse the effect of adropin alone but, rather, ameliorated all PTZ-induced changes and even prolonged the latencies of the first myoclonic jerk and GTCS, for which adropin alone was ineffective. These findings indicate that NO has a proconvulsant tendency in the model and PTZ dose we used. Moreover, L-NAME appears to potentiate the effects of adropin. However, previous studies demonstrated that L-NAME can exert both pro- and anti-convulsant effects in a context-dependent manner (Hendrickx et al. 2022; Paul and Subramanian 2002; Pramila et al. 2015). The lack of L-NAME administration alone in our study prevents direct assertion that it has an anticonvulsant effect in this study. However, our findings imply that the anticonvulsant effect of adropin likely involves a mechanism that is different from the nitric oxide system. The ability of L-NAME to enhance the effects of adropin necessitates further investigation to elucidate its mechanisms of action.

## Conclusion

Taken together, our results suggest that adropin demonstrates anticonvulsant actions through mitigating the behavioral epileptic seizures, neuroinflammation and oxidative stress. In addition, adropin exhibits its anti-neuroinflammatory effects through reducing the activation of NF-κB/IkB-α signaling pathway. Thus, adropine may represent an attractive alternative approach in the treatment of epileptic seizures in the future with its versatile effects.

## Limitations of the study

The mechanistic conclusions would be further supported if we performed direct protein quantification methods such as Western blotting or immunohistochemistry in addition to the ELISA method to measure biochemical markers in neuronal tissue homogenates. Because, although ELISA provides useful quantitative data, it does not allow for the assessment of specific protein isoforms, post-translational modifications, or subcellular localization-factors that are critical when interpreting signaling activity, particularly for phosphorylated forms of NF-κB and IκB-α.

## Data Availability

Data is provided within the manuscript.
